# *Streptococcus suis* surface-antigen recognition by antibodies and bacterial elimination is influenced by capsular polysaccharide structure

**DOI:** 10.3389/fcimb.2023.1228496

**Published:** 2023-07-21

**Authors:** Dominic Dolbec, Mélanie Lehoux, Masatoshi Okura, Daisuke Takamatsu, Marcelo Gottschalk, Mariela Segura

**Affiliations:** ^1^ Research Group on Infectious Diseases in Production Animals (GREMIP) and Swine and Poultry Infectious Diseases Research Center (CRIPA), Department of Pathology and Microbiology, Faculty of Veterinary Medicine, University of Montreal, Saint-Hyacinthe, QC, Canada; ^2^ Division of Transboundary Animal Disease Research, National Institute of Animal Health, National Agriculture and Food Research Organization, Kagoshima, Japan; ^3^ Division of Infectious Animal Disease Research, National Institute of Animal Health, National Agriculture and Food Research Organization, Tsukuba, Japan; ^4^ The United Graduate School of Veterinary Sciences, Gifu University, Gifu, Japan; ^5^ Joint Graduate School of Veterinary Sciences, Gifu University, Gifu, Japan

**Keywords:** *Streptococcus suis*, capsular polysaccharide, serotype, mouse, antibody

## Abstract

*Streptococcus suis* is an encapsulated bacterium causing severe diseases in swine. Here, we compared the protective properties of the capsular polysaccharide (CPS) of different *S. suis* serotypes by using serotype-switched mutants in a mouse model of infection. CPS structure influenced bacterial survival in mice, antibody binding, and antibody-mediated bacterial killing. The CPS of serotypes 3, 4 and 14 allowed more antibody binding and bacterial elimination than the CPS of serotypes 2, 7 and 9. Results suggest that the different CPS structures of *S. suis* provide varying levels of protection by influencing antigen availability and elimination by the host immune system.

## 1 Introduction

*Streptococcus suis* is an important Gram-positive encapsulated bacterium that mainly causes disease in weaned piglets (Gottschalk and Segura, 2019) and is also capable of zoonotic infections (Gottschalk et al., 2010). Pig farms around the globe are considered positive for *S. suis* as it is a natural inhabitant of the upper respiratory tract of swine (Higgins et al., 2013). In the presence of virulent strains, *S. suis* infection in pigs can lead to serious life-altering conditions such as septicemia leading to sudden death, meningitis, arthritis and endocarditis (Gottschalk and Segura, 2019). Prompt recognition of early clinical signs related to *S. suis* infection followed by treatment with appropriate antibiotics maximizes the survival of affected pigs (Gottschalk, 2020). However, prudent usage of antimicrobials is recommended as multiple strains of *S. suis* presenting antimicrobial resistances have been detected worldwide (Varela et al., 2013; Hadjirin et al., 2021; Uruen et al., 2022) and such strains represent health risks as possible antibiotic resistance reservoirs able to spread resistance genes to other pathogenic streptococci (Palmieri et al., 2011; Uruen et al., 2022). In this light, vaccines have become highly desirable to prevent *S. suis* infections on the farm and reduce antimicrobial usage. Multiple efforts have been made to develop efficacious vaccines against *S. suis*, however, there is currently no effective universal vaccine commercially available (Segura, 2015).

An important issue encountered in the development of a universal vaccine is the high genotypic and phenotypic diversity of *S. suis* isolates. Indeed, there are 29 distinct serotypes of *S. suis* that are defined by the immunogenicity of the capsular polysaccharide (CPS) that surrounds the bacteria (Okura et al., 2016). Additionally, significant differences within strains of a same serotype of *S. suis* have been uncovered by multilocus sequence typing (MLST) and finally, the distribution of serotypes and sequence types (ST) also highly differs between regions (Goyette-Desjardins et al., 2014) which makes the development of a universal cross-protective vaccine complicated. Most studies aimed to understand the immune response, protective antigens and vaccine development have focused on serotype 2 (Segura, 2015). Regarding the other serotypes of *S. suis*, studies remain scarce (Rieckmann et al., 2020). In the absence of efficacious commercial vaccines to prevent diseases caused by *S. suis*, the industry has resorted to the use of autogenous vaccines made by licensed laboratories. Autogenous vaccines are formulated with killed bacteria (bacterins) made from one or many *S. suis* strains isolated from the farm and are then only administered to the animals of that same farm with the objective to provide protection against the local circulating strains. The few controlled experimental studies on *S. suis* bacterins have shown contradictory results (Segura, 2015; Corsaut et al., 2020; Rieckmann et al., 2020), and there is no consistent evidence that bacterins providing protection against one serotype could provide protection against other strains and serotypes (Baums et al., 2009).

As there is still a lot of uncertainty regarding the efficacy of *S. suis* vaccines, more knowledge is needed to achieve better understanding of what influences cross-protection. One bacterial component that could influence such cross-protection would be the CPS that protects *S. suis*. Indeed, the CPS of *S. suis* serotype 2 is a known critical virulence factor implicated in many functions such as the impairment of phagocytosis and cytokine release by innate immune cells by masking bacterial surface components, such as proteins (Lecours et al., 2011). The biochemical structure of the CPS can highly differ between *S. suis* serotypes (Goyette-Desjardins et al., 2020) and differential exposure of cell wall components (mainly proteins) could influence host cell interactions and virulence (Okura et al., 2021). However, it remains unknown if these differences in the CPS between *S. suis* serotypes can influence the cross-protective potential of antibodies directed against sub-capsular antigens. In this study, serotype-switched mutants of *S. suis* were used to compare the effect of CPS structures of serotypes 2, 3, 4, 7, 8, 9 and 14 on antibody production, antibody binding and antibody-induced bacterial elimination. All the mutants possess the same sub-capsular antigens (those of the serotype 2 background) but express different CPS at their surfaces. By using this strategy, only the effect of the CPS on the antibody response against the same sub-capsular antigens was evaluated.

## 2 Methods

### 2.1 *S. suis* strains and growth conditions

The well characterized and highly virulent *S. suis* serotype 2 P1/7 strain (SS2), originally isolated from a pig with meningitis (Slater et al., 2003), was used along with previously described isogenic serotype-switched mutant strains SS2to3, SS2to4, SS2to7, SS2to8, SS2to9 and SS2to14 that respectively express the CPS of serotypes 3, 4, 7, 8, 9 and 14 (Okura et al., 2021). The serotype-switched mutants functionally possess and express the CPS of the donor serotype, as previously reported (Okura et al., 2021). In addition, a non-encapsulated isogenic mutant strain Δ*cpsF* was also included in the studies (Lecours et al., 2011). Bacterial culture and preparation were done as previously described (Auger et al., 2018).

### 2.2 Mouse experimental procedures

A well-standardized C57BL/7 mouse model of infection was used (Lecours et al., 2016). All mouse procedures, including euthanasia according to an established clinical endpoint grid to minimize the suffering of animals seriously affected by infections, were approved by the Animal Welfare Committee of the University of Montreal (Research protocols: Rech-1399 and Rech-1523). Four-week-old mice were acclimatized to laboratory conditions with free access to water and food pellets during a whole week before experiments were carried out. Mice were either injected with a sub-lethal dose of 1 x 10^6^ CFU of live SS2, or its isogenic serotype-switched mutants, by intraperitoneal injection on days 0 and day 14. For each experiment, negative-control mice injected with the matching vehicle solution were included. Mouse tail vein blood samples were taken 24 h post-infection, serially diluted in phosphate buffered saline (PBS) pH 7.4 and plated on Todd Hewitt Broth (THB, Burlington, ON, Canada) agar plates to determine bacterial blood burden. Serum samples were collected from the submandibular vein on day 14 post-infection and total sera were collected following euthanasia on day 28 post-infection. Serum samples were kept at -80°C for further analysis. Hyperimmunized sera was collected from a separate group of mice that were infected a total of 5 times with SS2 with each infection performed 7 days after the previous one and total sera collected a week after the last infection.

### 2.3 Titration of *S. suis* specific antibodies

The titers of anti-*S. suis* antibodies in infected mouse sera were determined as previously described (Goyette-Desjardins et al., 2020), with Polysorp MicroWell plates (Nunc-Immuno; Canadawide Scientific, Toronto, ON, Canada) coated with a bacterial suspension of whole SS2. In selected experiments, the non-encapsulated isogenic mutant strain Δ*cpsF* was used as antigen for the coating. Sera collected from mice infected with either SS2 or serotype-switched mutants were serially diluted and added to the wells. Peroxidase-conjugated detection antibodies allowed the detection of IgM or IgG antibody classes. All antibodies and dilutions used are listed in **Table 1**.

**Table 1:** Antibodies used.

*Target antigen*	*Target organism*	*Host organism*	*Clonality*	*Fluorochrome/conjugate*	*Dilution used*	*Application*	*Manufacturer*	*Catalog number*
IgM	Mouse	Goat	Polyclonal	HRP	1/1000	ELISA	Southern Biotech	1021-05
IgG	Mouse	Goat	Polyclonal	HRP	1/4000	ELISA	Jackson ImmunoResearch	115-035-071
IgG	Mouse	Goat	Polyclonal	AF647	1/100	FACS	Invitrogen	A-21235

### 2.4 Detection of antibody fixation on live bacteria using an hyperimmune serum against SS2

Detection of antibody fixation on live SS2 and its isogenic mutants SS2to3, SS2to4, SS2to7, SS2to8, SS2to9, SS2to14 and Δ*cpsF* was performed by flow cytometry. Bacterial suspensions containing 1x10^6^ CFU were distributed in microtubes and centrifuged. Bacterial pellets were resuspended in PBS supplemented with 2% fetal bovine serum (FBS; Gibco, Burlington, ON, Canada) along with 25 µl of SS2-hyperimmunized mouse sera and incubated at 4°C for 30 min. Following washing, pellets were resuspended in PBS-FBS containing anti-IgG-AF647 and incubated at 4°C for 30 min in the dark. Following staining, bacteria were washed, resuspended in PBS and immediately read on a BD LSRFortessa X-20 Cell Analyzer. Data collected was analyzed on FlowJo version 10. More information on the antibody and dilution used is listed in **Table 1**.

### 2.5 Antibody-dependent *in vitro* opsonophagocytosis assay

Antibody bactericidal assays were performed as described previously (Goyette-Desjardins et al., 2015). Briefly, blood from naive mice was mixed with serum collected from either naive mice (used as negative control – no killing observed) or mice infected twice with *S. suis* SS2 P1/7 strain. Blood-serum preparations were then put in contact with bacterial preparations of *S. suis* serotype 2 P1/7 strain or the isogenic serotype-switched mutants 2to3, 2to9 and 2to14. Samples were collected after 2 h of incubation to evaluate bacterial killing percentages as described (Goyette-Desjardins et al., 2015). The opsonophagocytosis (OPA) assay using whole blood cells (leukocytes) is considered a better approach than the use of a single cell type to measure antibody-dependent opsonophagocytosis killing of *S. suis*. OPA assays performed with phagocytic cell lines or purified cell types, underestimate the complexity of blood bactericidal activity (Goyette-Desjardins et al., 2015).

### 2.6 Statistical analysis

To evaluate statistical differences between two paired groups, parametric data were analyzed using a paired *t*-test and non-parametric data were analyzed using a Signed rank test. For unpaired groups, parametric data were analyzed using unpaired *t*-test and non-parametric data were analyzed using Mann-Whitney rank sum test. To evaluate statistical differences between three groups or more, parametric data were analyzed using one-way analysis of variance (ANOVA) followed by the Tukey Method. Non-parametric data were analyzed using Kruskal-Wallis One-Way ANOVA followed by Dunn’s Method. A *p* < 0.05 was considered as statistically significant. Statistical analyses were done using SigmaPlot version 11.0.

## 3 Results

### 3.1 Host control of bacterial burden and production of antigen-specific antibodies is affected by the capsular polysaccharide structure of *S. suis*

**Figure 1:**
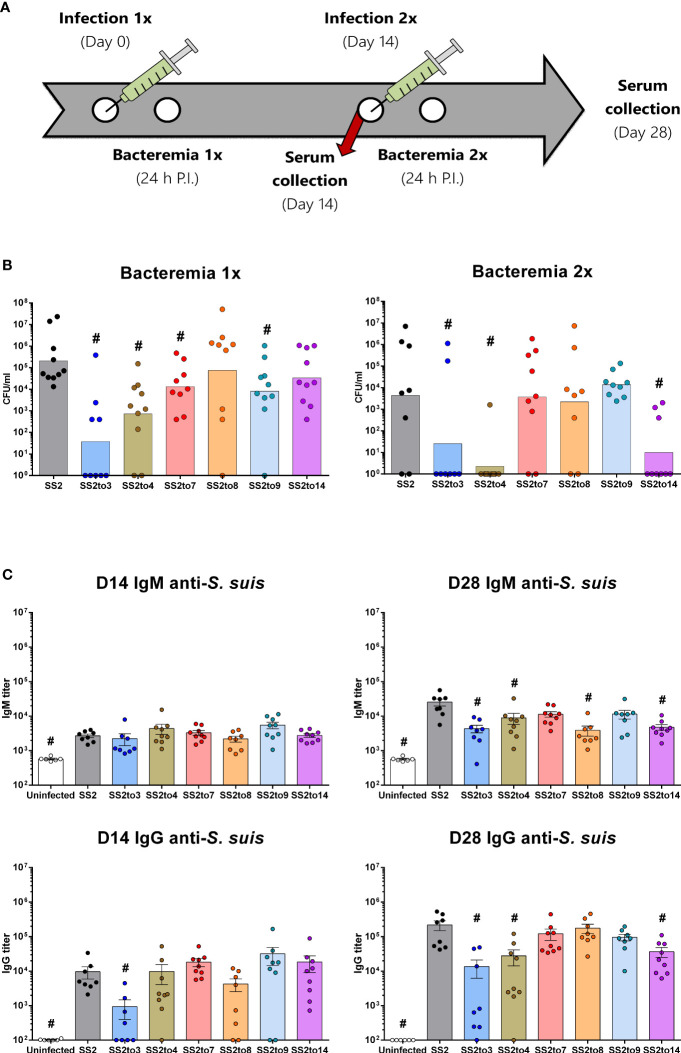
Bacterial control and antibody production is affected by the capsular polysaccharide structure of *Streptococcus suis*. Groups of C57BL/6 mice were infected with 1 x 10^6^ CFU doses of live wild-type *S. suis* serotype 2 strain P1/7 (SS2) or isogenic serotype-switched mutants expressing the capsular polysaccharide of serotypes 3 (SS2to3), 4 (SS2to4), 7 (SS2to7), 8 (SS2to8), 9 (SS2to9) or 14 (SS2to14). **(A)** Timeline of the experimental infections performed in the different groups of mice along with the bacteremia and sera collection post-infection (P.I.) sampling times. **(B)** Bacterial blood burden of mice 24 hours after the first (*n*; SS2 = 10, SS2to3 = 9, SS2to4 = 10, SS2to7 = 9, SS2to8 = 9, SS2to9 = 10 and SS2to14 = 10) and second (*n*; SS2 = 8, SS2to3 = 8, SS2to4 = 9, SS2to7 = 9, SS2to8 = 8, SS2to9 = 9 and SS2to14 = 9) *S. suis* infection. Data are represented as individual values with geometric means. **(C)** Anti-*S. suis* IgM and IgG titers of sera collected from mice on day 14 (*n*; Uninfected = 6, SS2 = 8, SS2to3 = 8, SS2to4 = 9, SS2to7 = 9, SS2to8 = 8, SS2to9 = 9 and SS2to14 = 9) and day 28 (*n*; Uninfected = 6, SS2 = 8, SS2to3 = 8, SS2to4 = 9, SS2to7 = 9, SS2to8 = 8, SS2to9 = 9 and SS2to14 = 9) measured by ELISA. Data are represented as individual values with mean ± SEM. ^#^indicated data significantly different (*p* < 0.05) with the SS2 strain as evaluated by Student’s *t*-test.

Groups of mice were infected with live SS2 or its isogenic serotype-switched mutants (**Figure 1A**). Monitoring of the bacterial blood burden of mice following the primary and secondary infections indicated that *S. suis* serotype-switched strains did not share a similar response *in vivo* (**Figure 1B**). Following the primary infection, mutants SS2to3, SS2to4, SS2to7 and SS2to9 had significantly lower bacterial blood burden loads than the SS2 strain while mutants SS2to8 and SS2to14 behaved similarly to SS2 wild-type strain. After a secondary infection, only the mutants SS2to3, SS2to4 still presented a significantly lower bacterial blood burden than the parent SS2 strain. In contrast to a primary infection, mutant SS2to14 showed impaired survival in blood after a secondary infection when compared to the parent strain.

Antibodies specific for *S. suis* SS2 (used as coating antigen) were evaluated 14 days following the primary and secondary infection (**Figure 1C**). Following the primary infection, IgM class production was similar between all groups of mice. Levels of anti-*S. suis* IgG were also similar amongst groups, except for mice infected with the mutant 2to3, which had significantly lower IgG antibody titers than the serotype 2 strain. Following the secondary infection, mice infected with mutants SS2to3, SS2to4, SS2to8 and SS2to14 had significantly lower IgM titers than the serotype 2 strain. For IgG class production, a similar profile was also observed: mutants SS2to3, SS2to4 and SS2to14, but not SS2to8, induced significantly lower levels of IgG than the serotype 2 strain. When the non-encapsulated mutant Δ*cpsF* was used as whole cell antigen in the ELISA, levels of IgG antibodies were similar than those obtained with wild-type SS2 strain as coating antigen, indicating that detected antibody titers are mostly against the sub-capsular antigens (**Supplemental Figure S1**).

### 3.2 The structure of *S. suis* capsular polysaccharide influences antibody binding and bacterial elimination

**Figure 2:**
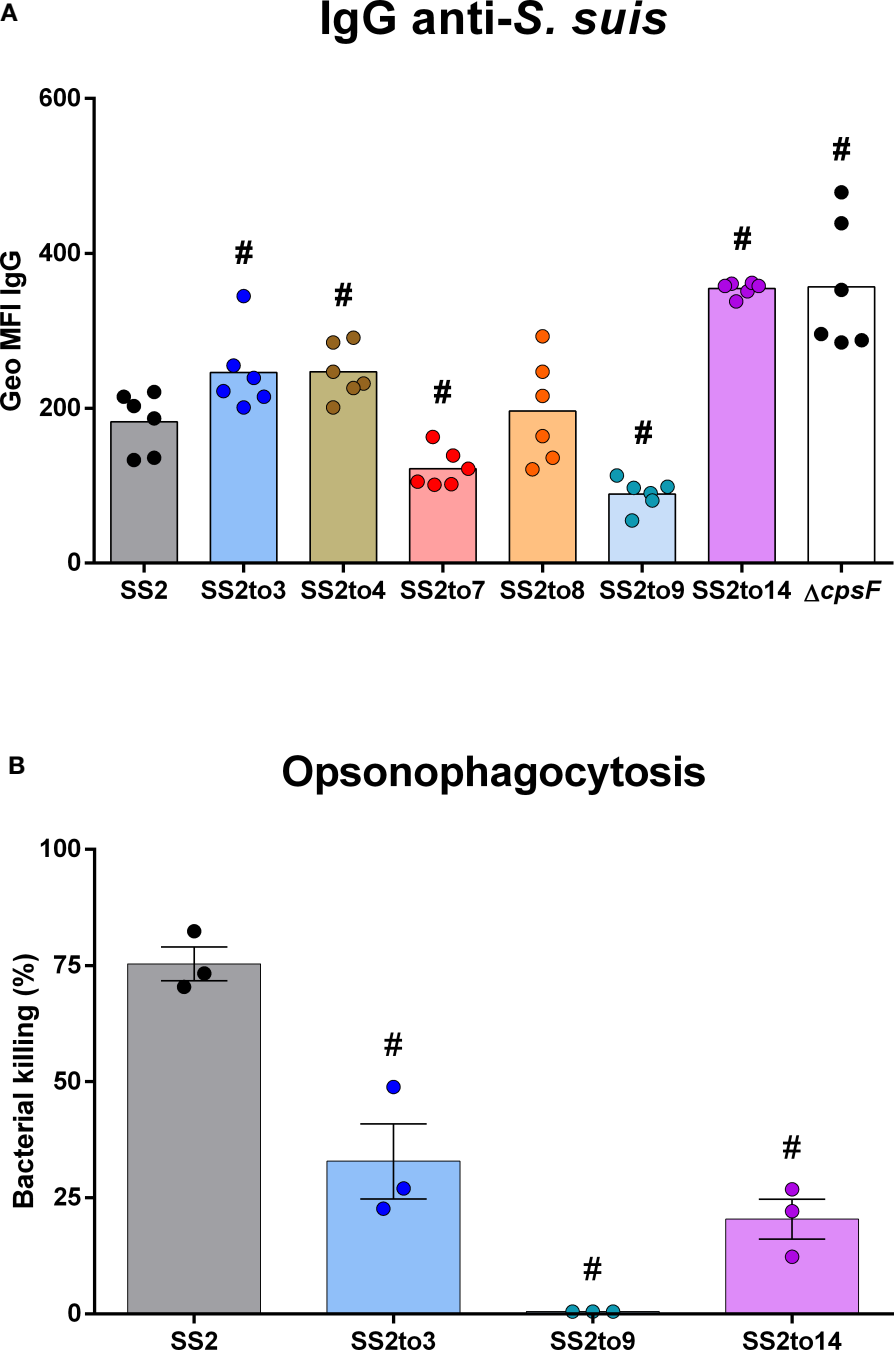
The structure of *Streptococcus suis* capsular polysaccharide influences antibody binding and bacterial elimination. The function of anti-*S. suis* antibodies in the sera of uninfected mice or mice hyperimmunized with *S. suis* were evaluated by flow cytometry and opsonophagocytosis killing assay (OPA). Binding of antibodies to *S. suis* serotype 2 strain P1/7 (SS2) or its isogenic mutants (SS2to3, SS2to4, SS2to7, SS2to8, SS2to9, SS2to14 and Δ*cpsF*) was evaluated by flow cytometry. **(A)** IgG Geo MFI (geometric mean fluorescence intensity) of different bacteria strains and mutants (*n*; SS2 = 6, SS2to3 = 6, SS2to4 = 6, SS2to7 = 6, SS2to8 = 6, SS2to9 = 6, SS2to14 = 6 and Δ*cpsF* = 6). Data are represented as individual geometric mean fluorescence intensity (Geo MFI) reads with mean. **(B)** Mouse serum-induced opsonophagocytosis killing of *S. suis*, evaluated by OPA for *S. suis* serotype 2 strain P1/7 (SS2) and isogenic serotype-switched mutants SS2to3, SS2to9 and SS2to14 (*n*; SS2 = 3, SS2to3 = 3, SS2to9 = 3 and SS2to14 = 3). Data are represented as individual values along with mean ± SEM. ^#^Indicated data significantly different (*p* < 0.05) with the SS2 strain as evaluated by Student’s *t*-test.

Flow cytometry was employed to visualize the levels of IgG class antibodies (from SS2-hyperimmunized mouse sera) able to bind to the cell wall components exposed at the bacterial surface (**Figure 2A**). As reported (Goyette-Desjardins et al., 2020), the hyperimmunized mouse sera contains mainly anti-proteins antibodies with negligible amounts of anti-CPS2 antibodies of the IgG class. Therefore, the assay detects IgG binding to the sub-capsular antigens of the different strains. Results showed that antibody binding was indeed influenced by the structure of the CPS. Mutants SS2to3, SS2to4, SS2to14 and the unencapsulated mutant Δ*cpsF* (used as control) bound significantly more IgGs than the SS2 strain. In contrast, mutants SS2to7 and SS2to9 showed significantly less bound IgGs. An OPA *in vivo* assay was used to evaluate antibody-mediated bacterial killing (**Figure 2B**). Results indicated that bacterial killing was affected by the structure of the CPS. As expected, sera of mice immunized twice with SS2 showed high killing percentages against a homologous challenge with the SS2 strain (used as control for the OPA test). Mutants SS2to14 (high IgG binding), SS2to3 (moderate IgG binding), and SS2to9 (low IgG binding) were selected for OPA analysis. Results showed that mutants SS2to3 and SS2to14 had equivalent killing percentages that were significantly higher than those of the SS2to9 mutant.

## 4 Discussion

The biochemical composition of the CPS that surrounds *S. suis* is known to be widely different between serotypes (Goyette-Desjardins et al., 2020). Our leading hypothesis was that these different CPS structures could differently influence the establishment and role of the antibody response against sub-capsular antigens (cell wall components). Strains expressing the CPS of serotypes 3 and 4 had markedly impaired survival in murine and porcine whole blood (Okura et al., 2021) and showed increased susceptibility to host defenses as they were more significantly eliminated than the parent SS2 strain during primary and secondary infections in mice. Albeit less pronounced, reduced blood bacterial burdens were also observed after primary infection with strains SS2to7 and SS2to9 in our mouse infection model (sub-lethal infection dose). However, the difference in bacteremia levels between these mutants and the parent SS2 strain was lost after a secondary infection and not noticeable in a high-dose mouse infection model (Okura et al., 2021). Altogether, these observations suggest that CPS structures of serotype 7 and 9 provides a similar protection against host defenses than that of CPS serotype 2. Interestingly, mutant SS2to14 behaves similarly than the parent strain SS2 after primary infection, as also reported in the high-dose mouse infection model (Okura et al., 2021). However, the capacity of this mutant to multiply in blood after a secondary infection was significantly impaired. This prompted us to evaluate the adaptive humoral response generated during infection, which has been known to be of importance for *S. suis* elimination (Holt et al., 1989; Blouin et al., 1994). Production of antibodies following a primary infection with *S. suis* was not affected by CPS structure, except for IgG production in mice infected with the CPS 3 mutant. We hypothesized that the quick elimination of the SS2to3 mutant *in vivo* (compared to SS2 and other serotype-switched mutants) might have reduced the quantity of bacterial antigens available to activate B cells and drive optimal IgG production. However, no clear correlation was found between IgG titers and bacterial blood burden (data not shown), although it is possible that the relatively low number of subjects used (*n* = 8-9) limited this analysis. Antibody production profiles were slightly different after the secondary infection where mice infected with CPS 3, 4 and 14 mutants had a significant reduction in anti-SS2 IgM and IgG production compared to the SS2 strain, which could again be attributed to less antigens available.

Protection from antibodies might be influenced by CPS structure as the CPS 14 mutant was significantly more eliminated in the presence of antibodies than the parent SS2 strain during a secondary infection. This observation came off as a surprise since mice infected with CPS 14 shared the same levels of antibodies against the bacteria than other strains after a primary infection (day 14). Enhanced elimination could be due to anti-CPS antibodies; however, anti-CPS titers are practically absent in animals infected with serotype 14 strains (Calzas et al., 2017). This observation led to the hypothesis that the different CPS structures might influence the ability of antibodies to bind to cell wall components exposed at the bacterial surface, which could then enable elimination by immune cells.

Flow cytometry experiments revealed that CPS 3, 4 and 14 mutants bound more IgG antibodies than the SS2 strain. This finding goes in hand with previous evidence that CPS 3, 4 and 14 mutants were more eliminated *in vivo* and would suggest that these CPS structures, and notably that of serotype 14, are more permeable to antibodies. On the other hand, CPS 7 and 9 conferred more protection against IgG binding than the parental CPS 2, which might explain the capacity of these strains to reach bacteremia levels similar to SS2 after the secondary infection. Taken together, these observations suggest that the different CPS structures of *S. suis* have different antigen masking properties, which could affect survival against the humoral response.

A controlled *in vitro* OPA using sera from mice infected with parent SS2 strain confirmed that the CPS structure could influence protection against antibodies directed against sub-capsular antigens. Since the serum used in the OPA was generated against SS2, any reaction with the serotype-switched mutants (expressing a different CPS) would be mainly directed against proteins. CPS mutants 3 and 14 were susceptible to antibody-mediated killing while the CPS 9 mutant showed resistance, indicating that CPS structures influence antigen availability and antibody detection which in turn influences bacterial elimination. It is worth mentioning that SS2 strain was significantly more eliminated than any of the tested strains in our OPA assay. This is because the sera used for all OPA trials was collected from a group of mice infected twice with the homologous SS2 strain and was positive for anti-CPS2 IgMs which are potent antibodies known to be able to induce high levels of opsonophagocytosis killing (Goyette-Desjardins et al., 2019). However, in the absence of specific anti-CPS IgMs, antibody-dependent phagocytosis (directed against sub-capsular antigens) of the mutant strains is affected by the CPS expressed at the bacterial surface.

The evidence gathered in this study indicates that the structural differences of *S. suis* CPS does influence the development and the role of the antibody response. Of the tested serotypes, the CPS of serotypes 2, 7, 8 and 9 provided better protection while the CPS of serotypes 3, 4 and 14 provided the weakest defense. It could be hypothesized that a more efficacious CPS structure would permit strains to persist in the host and cause disease more easily. This could help explain the overwhelming presence of serotype 2 strains in both pig and human cases world-wide (Goyette-Desjardins et al., 2014), and the rising incidence of serotype 9 strains, which are particularly virulent in European farms (Segura et al., 2020). However, serotype 3 strains are highly prevalent in the USA (Goyette-Desjardins et al., 2014) despite being equipped with a CPS that does not offer optimal protection and is also considered ‘‘highly’’ immunogenic (Goyette-Desjardins et al., 2020). Taking this into account, the ability of the CPS structure to influence antibody development and role is thus not solely responsible for virulence. Indeed, strain dependent differences can be observed within a same serotype (Auger et al., 2018) suggesting that other bacterial components might also play an important role in virulence.

There are currently over 60 published studies on *S. suis* vaccine candidates catalogued by PubMed, with some studies reporting efficacy against specific strains of *S. suis*. However, multiple serotypes of *S. suis* are present in herds (Reams et al., 1996) and mixed infections caused by different serotypes have been reported (Johannson, 2006). In this light, it would be ideal to develop a ‘‘universal’’ cell-surface protein vaccine that offers cross-protection against multiple strains and serotypes of *S. suis*. However, the results obtained in this study suggest that obtaining cross-protection might be more difficult than originally thought. Indeed, an “universal’’ vaccine made with highly conserved antigens shared between most serotypes and strains of *S. suis* could elicit strong antibody responses against said antigens but still fail to provide protection (Dumesnil et al., 2019; Weisse et al., 2021), probably due to the fact that CPS structures provide different levels of antigen masking and protection. As an indicator, autovaccines for *S. suis* serotype 9 have been found to be less efficacious than ones developed against the serotype 2 (Segura et al., 2020), which could be due to the higher level of bacterial protection conferred by the serotype 9 CPS. This study is the first experimental report that directly compares the influence of the CPS structure from multiple serotypes of *S. suis* on antibody production, antibody binding and antibody-induced bacterial killing. The results obtained indicate that CPS structure plays an important role in antigen availability and bacterial elimination, a finding of importance for vaccine development. Indeed, when anti-CPS specific antibodies are absent, the IgG anti-protein response would have a variable role depending on the capsular serotype of the strain. This study identifies the CPS of serotypes 2, 7, 8 and 9 as a potential high-risk since those CPS confer higher levels of protection against antibodies. Overall, this study highlights the need to closely monitor cross-protection when designing *S. suis* vaccines.

## Data availability statement

The raw data supporting the conclusions of this article will be made available by the authors, without undue reservation.

## Ethics statement

The animal study was reviewed and approved by Animal Welfare Committee of the University of Montreal.

## Author contributions

DD and MS conceived the study. DD and ML performed all animal infections and sampling. DD analyzed the animal data. DD performed the ELISA and OPA experiments. ML performed the FACS experiments and DD analyzed the FACS data. DD performed statistical analyses. DD prepared tables and figures. DD and MS interpreted the data. DD and MS wrote the paper. MO and DT provided the mutants. All authors read and approved the final manuscript.
