# Supplementary material for: Streptococcus suis surface-antigen recognition by antibodies and bacterial elimination is influenced by capsular polysaccharide structure
Source: Front Cell Infect Microbiol. 2023 Jul 21;13:1228496. doi: 10.3389/fcimb.2023.1228496 (PMC10401424; doi:10.3389/fcimb.2023.1228496)
Supplement: Supplementary file 1 [file Image_1.pdf]

Supplementary Material

Streptococcus suis surface-antigen recognition by antibodies and bacterial elimination is influenced by capsular polysaccharide structure

Dominic Dolbec[1], Mélanie Lehoux[1], Masatoshi Okura[2], Daisuke Takamatsu[3, 4, 5], Marcelo Gottschalk[1], and Mariela Segura[1*]

[1]Research Group on Infectious Diseases in Production Animals (GREMIP) and Swine and Poultry Infectious Diseases Research Center (CRIPA), Department of Pathology and Microbiology, Faculty of Veterinary Medicine, University of Montreal, Saint-Hyacinthe, Quebec, Canada

[2]Division of Transboundary Animal Disease Research, National Institute of Animal Health, National Agriculture and Food Research Organization, Kagoshima, Kagoshima, Japan

[3]Division of Infectious Animal Disease Research, National Institute of Animal Health, National Agriculture and Food Research Organization, Tsukuba, Ibaraki, Japan

[4]The United Graduate School of Veterinary Sciences, Gifu University, Gifu, Gifu, Japan

[5]Joint Graduate School of Veterinary Sciences, Gifu University, Gifu, Gifu, Japan

*** Correspondence:** Corresponding Author: mariela.segura@umontreal.ca

Anti-*S. suis* IgG antibodies are mostly directed against the sub-capsular antigens

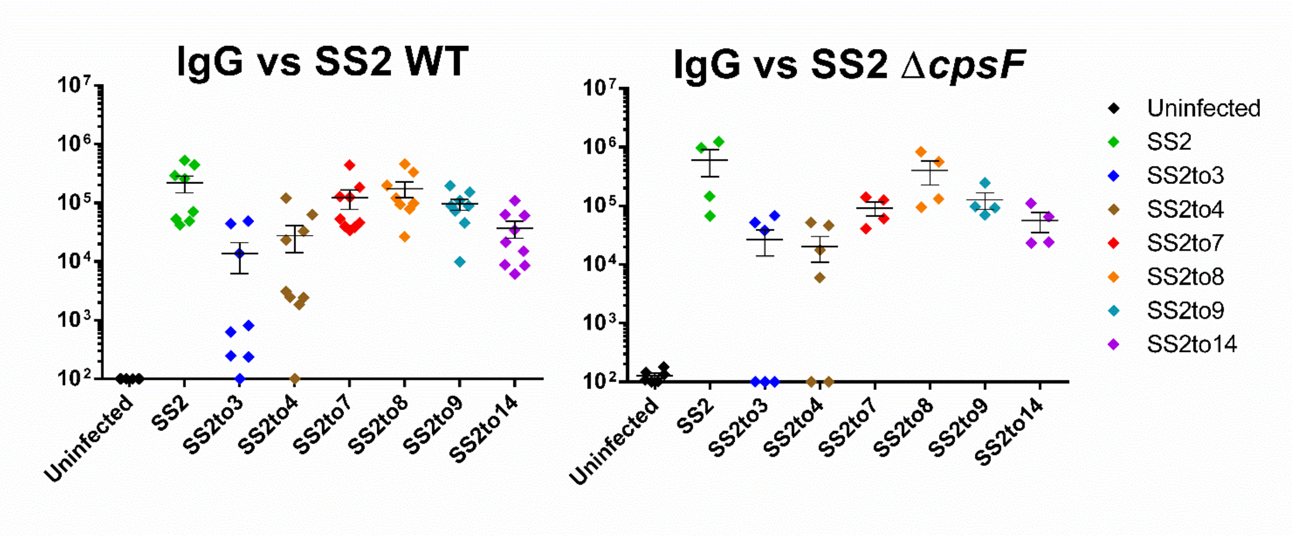

Supplementary Figure S1. Groups of C57BL/6 mice were infected with 1 x 10^6 CFU doses of live wild-type *S. suis* serotype 2 strain P1/7 (SS2) or isogenic serotype-switched mutants expressing the capsular polysaccharide of serotypes 3 (SS2to3), 4 (SS2to4), 7 (SS2to7), 8 (SS2to8), 9 (SS2to9) or 14 (SS2to14). Anti-*S. suis* IgG titers of sera collected from mice on day 28 measured by ELISA against whole SS2 wild-type (WT) bacteria (*n*; Uninfected = 6, SS2 = 8, SS2to3 = 8, SS2to4 = 9, SS2to7 = 9, SS2to8 = 8, SS2to9 = 9 and SS2to14 = 9) or SS2 Δ*cpsF* bacteria (*n*; Uninfected = 6, SS2 = 4, SS2to3 = 6, SS2to4 = 6, SS2to7 = 4, SS2to8 = 4, SS2to9 = 4 and SS2to14 = 4).
